# Developing IntegRATE: a fast and frugal patient-reported measure of integration in health care delivery

**DOI:** 10.5334/ijic.1597

**Published:** 2015-03-27

**Authors:** Glyn Elwyn, Rachel Thompson, Roshen John, Stuart W Grande

**Affiliations:** The Dartmouth Center for Health Care Delivery Science, The Dartmouth Institute for Health Policy and Clinical Practice, Dartmouth College, Hanover, NH, USA; The Dartmouth Institute for Health Policy and Clinical Practice, Dartmouth College, Hanover, NH, USA; The Dartmouth Center for Health Care Delivery Science, Dartmouth College, Hanover, NH, USA; The Dartmouth Center for Health Care Delivery Science, Dartmouth College, Hanover, NH, USA

**Keywords:** integration of care, care coordination, continuity of care, measurement, patient-reported measures, cognitive interviews

## Abstract

**Background:**

Efforts have been made to measure integration in health care delivery, but few existing instruments have adopted a patient perspective, and none is sufficiently generic and brief for administration at scale. We sought to develop a brief and generic patient-reported measure of integration in health care delivery.

**Methods:**

Drawing on both existing conceptualisations of integrated care and research on patients’ perspectives, we chose to focus on four distinct domains of integration: *information sharing*, *consistent advice*, *mutual respect* and *role clarity*. We formulated candidate items and conducted cognitive interviews with end users to further develop and refine the items. We then pilot-tested the measure.

**Results:**

Four rounds of cognitive interviews were conducted (*n* = 14) and resulted in a four-item measure that was both relevant and understandable to end users. The pilot administration of the measure (*n* = 15) further confirmed the relevance and interpretability of items and demonstrated that the measure could be completed in less than one minute.

**Conclusions:**

This new measure, IntegRATE, represents a patient-reported measure of integration in health care delivery that is conducive to use in both routine performance monitoring and research. The psychometric properties of the measure will be assessed in the next stage of development.

## Introduction

Managing illness is difficult enough without having to navigate poorly integrated health care delivery systems. Yet many patients report that this is precisely the challenge they face [1]. Patients *expect* their care to be provided by teams of well-integrated health professionals [2] but research shows that many patients experience fragmentation, poor coordination, lack of information and confusion [3–5]. Patients also face the cost and burden of attending multiple clinics, often with marginal benefit, as well as duplication of effort, poor communication and conflicting advice [6, 7]. Given the increasing number of people with multiple long-term conditions requiring ongoing, coordinated care [8], the lack of integration in health care delivery has become one of the most urgent problems facing both systems and patients. Moreover, a recent bibliometric analysis demonstrated that the need to provide integrated care for patients has become a central health policy concern [9].

Routinely monitoring integration in health care delivery from the patient perspective is a critical step in understanding and improving both the patient experience and the quality and efficiency of health care. Yet, two key factors have impeded progress towards this goal. First, there has long been a lack of conceptual clarity in the field of integration in health care delivery [10–12]. A multitude of terms including *integration of care*, *care coordination*, *continuity of care*, *collaborative care* and *joined-up care* have been used, often interchangeably, generating confusion and ambiguity. At the same time, many competing frameworks and models of integration have been proposed. While most are multidimensional, they differ in emphasis from the seamlessness of care provision within a single health care setting to the effectiveness of communication and cooperation across a multi-site network of providers or the continuous provision of care by the same individual health care provider [13, 14]. Second, researchers have largely failed to approach the issue of integration in health care delivery in a patient-centric way [10]. Historically, the dominant perspective underlying conceptualisations of integration has been top-down rather than bottom-up, giving rise to both frameworks and assessment approaches that may not emphasise concepts that reflect the problems that patients commonly experience.

In spite of the barriers described above, recent years have seen a burgeoning of instruments developed to measure integration in health care delivery (and equivalent concepts) from the patient perspective. Uijen and colleagues undertook a systematic review of instruments published between 2005 and 2011, identifying 17 patient-reported measures [15]. Our recent search of the subsequently published literature identified three more patient-reported measures [16–18], for a total of 20 measures. However, notwithstanding their value for use in research, these existing measures have limited utility for the routine monitoring of integration in health care delivery. Sixteen of the 20 measures were created for patients with specific clinical conditions or in specific care settings. The specificity of these measures undermines their wide applicability and precludes their use for cross-institutional comparison and learning. Additionally, many of the measures have a significant number of items and appear unnecessarily burdensome to patients, despite growing evidence that brief health-related measures can perform as well as more comprehensive measures [19]. The average number of items in the reviewed measures was 31, with a range of 12–129, hindering their administration at scale.

Given the limitations of current measures, we sought to develop a new patient-reported measure of integration in health care delivery in partnership with end users. Our specific objective was to develop an instrument that is brief, generic, valid and reliable, and that assesses aspects of health care delivery that are both salient to patients and readily modifiable. We intended for the measure to be suitable for all individuals who have recently interacted with two or more people while receiving health care, whether those people were members of an identified health care team within a single setting or members of a multi-site provider network. In this paper, we describe the process of developing and refining survey items through a series of cognitive interviews, an established method to ensure item interpretability [20], and verifying the interpretability of the items through a pilot administration of the final instrument.

## Theory and methods

### Phase 0: preliminary development

#### Domain development

Drawing on both existing conceptualisations of integrated care [13–15] and the findings of research examining patients’ experiences and perspectives [7, 21–24), we chose to focus on four distinct domains of integration. The first domain, *information sharing*, represents effective information transfer across members of the health care team. The second domain, *consistent advice*, represents concordant information provision by members of the health care team. The third domain, *mutual* *respect*, represents respect and collaboration among members of the health care team. The final domain, *role clarity*, represents patient understanding of the different roles of the various members of their health care team. These domains are not specific either to integration within an identified health care team in a single setting or to integration of those working in a network across multiple settings.

#### Candidate item formulation

We formulated a pool of candidate items for each domain, focusing purposefully on negatively worded items that assessed the frequency of *poor* care integration, based on the premise that patients may more readily recall examples of poor quality care. For *information sharing*, we focused on items assessing how often the patient was required to do otherwise unnecessary or additional work because of a deficit in information transfer by members of the health care team. For *consistent advice*, we focused on items assessing how often the patient experienced confusion due to receipt of conflicting information or advice. For *respect*, we focused on items assessing how often the patient experienced discomfort because of a demonstrated lack of mutual respect or collaboration among team members. For *role clarity*, we focused on items assessing how often the patient lacked awareness of which team member to contact in the event of a question or concern.

#### Opening statement development

We developed an opening statement to provide guidance to respondents completing the measure. The opening statement oriented respondents both to the scope of the health care delivery team undergoing evaluation, and the intended timeframe when responding to the questions. The opening statement read:

Receiving health care often means seeing different people, such as office staff, nurses, doctors, and other health professionals. Please think about a health issue that has led you to see different people over the last few weeks or months and answer the following questions:

### Phase 1: cognitive interviews

#### Participants

Participants were adults recruited from non-clinical areas of a large tertiary hospital in the Northeastern USA. Although participants were required to be confident reading and writing in English, no other eligibility criteria were applied. We aimed to recruit a sample diverse in age with approximately equal numbers of men and women. Rather than predetermine the sample size, we aimed to continue recruitment within each interview round until saturation was reached [20].

#### Procedure

One of two interviewers (RJ, SG) approached prospective participants and sought their informed consent to participate in the cognitive interviews. Participants received a written copy of the candidate items. The interviewer first asked participants to read and react to the candidate items. Next, the interviewer asked participants to describe their overall comprehension of the items by explaining the meaning of each item in their own words. The interviewer used a set of prompts to probe participants further about their understanding or interpretation of the items. Finally, participants completed a brief demographic survey. Each participant received monetary compensation for his or her time.

All interviews were recorded and summarised by the interviewer. After each round of interviews, we refined the candidate items based on participant reactions and feedback. We continued conducting interviews until saturation was reached and we had arrived at a final set of items that participants easily understood and interpreted consistently and correctly.

### Phase 2: pilot administration

#### Participants

Again, participants were adults recruited from non-clinical areas of a large tertiary hospital in the Northeastern USA. Although participants were required to be confident reading and writing in English, no other eligibility criteria were applied. We aimed to recruit a sample of approximately 15 adults.

#### Procedure

One interviewer (SG) approached prospective participants and sought their informed consent to participate in the pilot test. Participants were provided with a written copy of the measure developed in Phase 1 and asked to complete it with respect to a previous care experience. For the pilot administration, we used four response options for each item: *Never*, *A little*, *A lot* and *Always*. The interviewer asked each participant to offer impressions of the items based on comprehension, ease of use and salience to their experiences. We also assessed the time taken to complete the instrument. Finally, participants completed a brief demographic survey. Each participant received monetary compensation for his or her time.

The research was reviewed by the Committee for the Protection of Human Subjects at Dartmouth College and deemed exempt from further review, and institutional permission for recruitment was provided.

## Results

### Cognitive interviews

A total of 14 participants (6 women and 8 men) were interviewed in four rounds. Participants had a median age of 46 years (range: 19–72 years). The majority of participants did not have a university degree (*n* = 8). Most participants identified as White (*n* = 9) or Asian (*n* = 4), and all identified as Not Hispanic or Latino. All participants but one spoke only English at home.

Table 1 displays the original candidate items and their iterative development over time, as well as the number of individuals interviewed in each round of cognitive interviews.

#### Item 1: information sharing

The initial candidate items assessing *information sharing* required two changes. First, participants considered the phrase ‘more’ redundant. Second, participants felt the phase ‘work well together’ was considerably more ambiguous than ‘share information with each other’. One participant offered the thought that:

… people who share information make things go smoother and faster, which further indicates how people work well together. (male, age 65)

Interviewees, when asked to express the meaning of this item in their own words, consistently exhibited good understanding of the underlying idea that poor informational continuity gave rise to inefficiency and required them to spend unnecessary time or effort compensating for deficits in information transfer.

#### Item 2: consistent advice

The item development for the domain of *consistent advice* focused in part on whether the use of the word ‘confused’ was better understood and more meaningful than the descriptive phrases ‘did not know what to do’ or ‘unsure what to do’. The idea of being ‘confused’ resonated more strongly. According to one participant:

‘confused’ strikes at the point where patients are caught being unsure of themselves and must recognize this is when they need to ask questions. (male, age 24)

We also assessed participants’ understanding of, and preferences for, the terms ‘conflicting’ versus ‘different’. Participants easily understood the phrase ‘conflicting information or advice’ and felt that its greater specificity was helpful. One participant's interpretation demonstrated the alignment in their understanding of the item:

one [person] is giving me one thing and another person is giving me another. (male, age 65).

By the third round, it was clear that the salient concern of patients was precisely related to being confused by conflicting advice and we therefore adopted this phrasing in the final item.

#### Item 3: mutual respect

The item constructed to assess *mutual respect* proposed alternative phrases about the degree to which observable conflict between team members had a negative effect on patients’ sense of well-integrated care. During the second round of interviews, we added the phrase ‘lose confidence’ to the existing candidates of ‘worried’, ‘concerned’ or ‘uncomfortable’. One participant seemed to capture the overall sentiment of responses saying:

Worried – not the best word to describe losing trust or confidence because of a lack of respect among providers. (male, age 49)

By the fourth round, it was evident that the word ‘uncomfortable’ best conveyed the sense of unease felt by participants when they witnessed conflict between team members or heard team members criticise each other. The word ‘concern’ seemed to refer to concern about other people, as suggested by this participant:

I'm sure people want to talk more about how they're feeling comfortable or uncomfortable rather being concerned for someone else… (female, 19)

Additionally, it became clear that the phrase ‘get along with’ was considerably easier to interpret than the word ‘cooperate’. As one participant said:

I would think that if they get along with each other, then they wouldn't be fighting, if you know what I'm saying – like cooperating with each other, you know what I'm saying. (male, age 57)

#### Item 4: role clarity

The item constructed to assess *role clarity* initially focused on the issue of ‘knowing who to’ talk to about a ‘question or concern’ or ‘health issue’. During the first two sets of interviews, participants seemed to interpret this in a general sense, stating that they would seek advice from their family doctor or other generalist. This was not aligned with the intent of the item, which was to assess how often the patient lacked awareness of which team member to contact in the event of a question or concern. Using the term ‘know’ led to expectations of having specific ‘knowledge’ and was considered unrealistic by participants:

I would leave that to the health care provider, because I wouldn't really know…, perhaps a health care provider should be the one telling you who to talk to?

(male, age 57)

In the fourth round we confirmed that the most accurately understood phrase referred to being ‘unclear whose job it was to deal with a specific question or concern’. Being ‘unclear’ was viewed as related to:

… information not provided effectively. (female, age 53)

The final item ‘How often were you unclear whose job it was to deal with a specific question or concern?’ was perceived positively by a participant who noted its salience:

… just being here, and all the stuff we've been through [today], this is a great question. (male, age 46)

### Pilot administration

A total of 15 participants (7 women and 8 men) completed the pilot test. Participants had a median age of 54 years (range: 19–75 years). The majority of participants did not have a university degree (*n* = 10). Most participants (*n* = 13) spoke only English at home, with the remaining two participants speaking both English and at least one other language. The majority of participants (*n* = 14) identified as White, while one participant identified as Asian, and all identified as Not Hispanic or Latino.

In the pilot administration, one person had difficulty with the first item but was able to comprehend the question on her second reading. Each participant was able to complete the four items in less than one minute. All participants commented that the questions were highly relevant to patients, and that integration of care was a serious concern, especially for people with multiple or chronic conditions. Further, those interviewed said these were:

… problems that patients like me have experienced. (male, age 57)

and that the questions addressed:

… things that as a patient I have to deal with all the time. (female, age 42)

## Discussion

The outcome of this study is ‘IntegRATE’, a new patient-reported measure of integration in health care delivery, developed through iterative rounds of cognitive interviews with end users. IntegRATE comprises four items which map onto the unique domains of *information sharing*, *consistent advice*, *mutual respect* and *role clarity*, assessing aspects of health care delivery that are both meaningful to patients and readily modifiable. The instrument is brief and can be completed in less than one minute, maximising its suitability for use in routine performance monitoring in health care, as well as in research. The instrument is also sufficiently generic to allow completion by any individual who has recently interacted with two or more people while receiving care for a health issue, whether those people are members of an identified health care team within a single setting or members of a multi-site network of providers.

In 2010, Uijen called attention to the need to measure integration in health care delivery from the patient's perspective [25]. Our preliminary work found that, despite a burgeoning of patient-reported measures of integration in recent years, existing instruments were typically lengthy and created for patients with specific conditions. These limitations of existing instruments led us to develop this new patient-reported measure. Strengths of our development process include a thorough review of existing approaches to assessment in the field and the identification of clearly articulated measurement domains. While these domains may not constitute a definitive conceptual model of integration in health care delivery, they comprise aspects of care frequently reported by patients to be important [7, 24), and are aligned with domains commonly assessed in other measures of integration [15, 18].

A further strength of our development process was the use of cognitive interviewing, a robust and widely advocated method for the development and refinement of survey items. At the same time, both the nature of the cognitive interview methodology adopted and the study setting resulted in considerable homogeneity in our sample of participants. Accordingly, we are cautious in generalising findings of the interpretability of the final items to the broader population. We will address this weakness during the next stages of development, which will involve testing the wider applicability and psychometric properties of the measure in more diverse populations, both under hypothetical conditions and in a broad range of clinical settings.

In all, this paper describes the first stage of work in the development and validation of IntegRATE, a patient-reported measure of integration in health care delivery. Compared with other tools [15], IntegRATE is brief, widely applicable and, thus, highly conducive to use in both performance monitoring and research. The next stages of development comprise the psychometric validation of the instrument and pilot implementation in routine practice.

## Figures and Tables

**Table 1.**
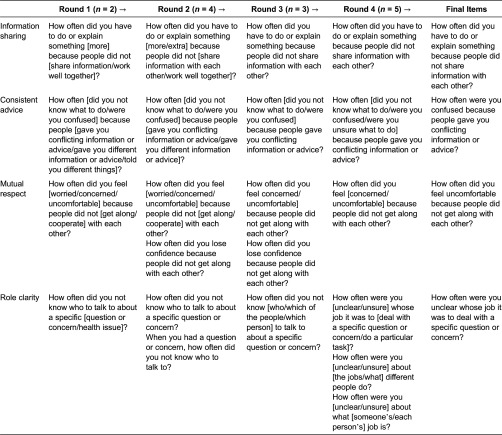
Candidate items tested in each round of cognitive interviews and final items.
